# The impact of COVID-19 on the development and consolidation of telemedicine

**DOI:** 10.1590/1516-3180.2021.139305042021

**Published:** 2021-05-10

**Authors:** Alessandro Wasum Mariani, Paulo Manuel Pêgo-Fernandes

**Affiliations:** I MD, PhD. Attending Surgeon, Thoracic Surgery Program, Instituto do Coracao, Hospital das Clinicas HCFMUSP, Faculdade de Medicina, Universidade de Sao Paulo, Sao Paulo, SP, BR.; II MD, PhD. Full Professor, Thoracic Surgery Program, Instituto do Coracao, Hospital das Clinicas HCFMUSP, Faculdade de Medicina, Universidade de Sao Paulo, Sao Paulo, SP, BR

The pandemic caused by coronavirus 19 (COVID-19) has given rise to a series of unprecedented challenges to various healthcare systems around the world.^1^ The solutions that have been found for many of these challenges may become definitive in certain scenarios. One very significant example of this is telemedicine, which has been defined by Maldonado et al.^2^ as: “use of information and communication technologies within healthcare to enable provision of healthcare-related services (expansion of attendance and coverage), especially in cases in which distance is a critical factor.”

Telemedicine particularly encompasses the following concepts:

- Teleconsultation: a form of attendance in which the doctor and the patient are in different places, done by means of audio or video equipment in which exchanges of information take place in real time. The biggest challenges regarding this are the lack of physical examination and problems relating to privacy.
- Telediagnosis: this consists of remote access to a given examination and issuing of reports on this, activated by a doctor at a distance. This is much used today within radiology. The main challenge is technical, especially because of the need for a broadband connection due to the large size of the files.
- Teleinterconsultation: exchange of information between professionals, at a distance. This is much used when an opinion from a certain specialist is desired. It may take place during a consultation in which a generalist is present with the patient and the specialist is at a distance. The main challenge regarding this relates to the privacy of information.
- Telesurgery: operations conducted by robotic equipment that is commanded remotely by a surgeon. In the past, the American government took interest in this for military use. The biggest challenges relate to the cost of the robotic equipment and to guaranteeing a stable connection. Because of these technical restrictions, its use is still practically entirely experimental.
- Teletriage: this consists of brief assessment of patients’ complaints through a telephone call, with referral for attendance when necessary.
- Telemonitoring: this refers to remote follow-up of patients’ state of health. It takes place through analysis on data and images from equipment used by the patient.

In Brazil, telemedicine was regulated by the Federal Medical Council (Conselho Federal de Medicina, CFM) in 2002, through resolution no. 1643. However, this resolution did not bring complete definition or detailing of a variety of points relating to this topic. Consequently, a second resolution that would adapt and permit implementation of this form of medical attendance was published in 2018. However, because of a large number of controversial issues, the 2018 resolution from the CFM was revoked in 2019.^3^

The COVID-19 pandemic then brought about a major change in Brazil. On April 15, 2020, law no. 13,989 was published in the Federal Official Gazette (Diário Oficial da União). Article 1 of this law states: “This law authorizes the use of telemedicine for as long as the crisis caused by coronavirus (SARS-CoV-2) continues.” From this time onwards, doctors and health-related companies started to implement a variety of aspects of telemedicine, especially with regard to teleconsultation.

The real impact of this measure, as well as its duration, remains to be defined. Nonetheless, on the basis of articles that have recently been published, the impression given is that telemedicine is here to stay, both in Brazil and worldwide.

Researchers at Duke University^4^ analyzed the adoption of telemedicine by different specialties in three different periods: before the pandemic, at the peak of the pandemic and after the reduction in the number of cases. The rate of adoption of telemedicine varied greatly among specialties: 3.2% for dermatology (the lowest) to 98.3% for psychiatry (the highest). In addition, differences regarding the profile of users were seen: Afro-American and male patients were less likely to use telemedicine than were white and female patients. The analysis on the three periods showed that the phase of the pandemic with the highest number of cases was the time when telemedicine was most used.

Chu et al.^5^ analyzed data on adoption of telemedicine before and during the pandemic, comparing rural and urban areas of Ontario. Before the pandemic, the use of teleconsultation was consistently low in both populations: 11 attendances per 1,000 rural patients versus 7 attendances per 1,000 urban patients (December 2019). During the pandemic (June 2020), there were increases in the use of telemedicine in both populations, but growth was higher in the urban area, to 220 visits per 1,000, versus 147 visits per 1,000 in the rural area. In relation to sex, 54.6% of the users were female, versus 45.4% who were male. These authors came to the conclusion that different strategies for promoting telemedicine might be necessary for populations living in different areas.

In relation to different specialties, a profusion of articles has been published, analyzing specific matters such as adaptation of telemedicine for use in different fields like dermatology,^6^ neurosurgery,^7^ cardiology^8^ and allergology,^9^ among others. Almost all of them come to similar conclusions: that telemedicine has become an important tool during the period of the pandemic and that its development should continue after the pandemic.

Nittari et al.^10^ published a review on the ethical aspects of telemedicine. Through analysis on a diversity of articles, they concluded that the main ethical concerns were the following: data privacy; the need to guarantee that the equipment is in good working order; the ever-faster obsolescence of technology; the need for professionals to have adequate training for attending patients in a virtual environment; and the lack of legal regulation that exists in most countries. Lastly, these authors concluded that if these concerns failed to be correctly addressed, a boomerang effect might be seen: after an exponential increase in the use of telemedicine due to the current situation of the pandemic, there would then be rapid regression afterwards.

Nine years have passed by since we last wrote an editorial on this subject.^11^ Since then, technology has advanced in an extraordinary manner. Nonetheless, it is certain that nothing has pushed forward the adoption of telemedicine more than the situation of the COVID-19 pandemic. We believe that this form of medical attendance is here to stay, but that it is still too early to predict what the definitive position of telemedicine will be after the COVID-19 pandemic.
